# Dysfunction of the NMDA Receptor in the Pathophysiology of Schizophrenia and/or the Pathomechanisms of Treatment-Resistant Schizophrenia

**DOI:** 10.3390/biom14091128

**Published:** 2024-09-06

**Authors:** Ruri Okubo, Motohiro Okada, Eishi Motomura

**Affiliations:** Department of Neuropsychiatry, Division of Neuroscience, Graduate School of Medicine, Mie University, Tsu 514-8507, Japan; okubo-r@med.mie-u.ac.jp (R.O.); motomura@clin.medic.mie-u.ac.jp (E.M.)

**Keywords:** schizophrenia, pathophysiology, pathomechanism, clozapine, NMDA/glutamate receptor, antipsychotics

## Abstract

For several decades, the dopamine hypothesis contributed to the discovery of numerous typical and atypical antipsychotics and was the sole hypothesis for the pathophysiology of schizophrenia. However, neither typical nor atypical antipsychotics, other than clozapine, have been effective in addressing negative symptoms and cognitive impairments, which are indices for the prognostic and disability outcomes of schizophrenia. Following the development of atypical antipsychotics, the therapeutic targets for antipsychotics expanded beyond the blockade of dopamine D2 and serotonin 5-HT2A receptors to explore the partial agonism of the D2 receptor and the modulation of new targets, such as D3, 5-HT1A, 5-HT7, and metabotropic glutamate receptors. Despite these efforts, to date, psychiatry has not successfully developed antipsychotics with antipsychotic properties proven to be superior to those of clozapine. The glutamate hypothesis, another hypothesis regarding the pathophysiology/pathomechanism of schizophrenia, was proposed based on clinical findings that N-methyl-D-aspartate glutamate receptor (NMDAR) antagonists, such as phencyclidine and ketamine, induce schizophrenia-like psychotic episodes. Large-scale genome-wide association studies (GWASs) revealed that approximately 30% of the risk genes for schizophrenia (the total number was over one hundred) encode proteins associated with glutamatergic transmission. These findings supported the validation of the glutamate hypothesis, which was inspired by the clinical findings regarding NMDAR antagonists. Additionally, these clinical and genetic findings suggest that schizophrenia is possibly a syndrome with complicated pathomechanisms that are affected by multiple biological and genetic vulnerabilities. The glutamate hypothesis has been the most extensively investigated pathophysiology/pathomechanism hypothesis, other than the dopamine hypothesis. Studies have revealed the possibility that functional abnormalities of the NMDAR play important roles in the pathophysiology/pathomechanism of schizophrenia. However, no antipsychotics derived from the glutamatergic hypothesis have yet been approved for the treatment of schizophrenia or treatment-resistant schizophrenia. Considering the increasing evidence supporting the potential pro-cognitive effects of glutamatergic agents and the lack of sufficient medications to treat the cognitive impairments associated with schizophrenia, these previous setbacks cannot preclude research into potential novel glutamate modulators. Given this background, to emphasize the importance of the dysfunction of the NMDAR in the pathomechanism and/or pathophysiology of schizophrenia, this review introduces the increasing findings on the functional abnormalities in glutamatergic transmission associated with the NMDAR.

## 1. Introduction

Antipsychotic medication for schizophrenia is one of the most successful therapies in psychiatry. Despite these efforts, based on analyses of regional and national prevalence, disability-adjusted life years (DALYs), and years lived with disability (YLDs) for numerous diseases/disorders from 1990 to 2019, the Global Burden of Disease Study 2019 ranked schizophrenia 20th among the top 25 leading causes of YLDs (depressive and anxiety disorders were ranked 2nd and 8th, respectively). The age-standardized DALY rate among overall mental disorders was over 12% [1]. These data raise the concern that, despite the successful development of numerous antipsychotics, these efforts in psychiatry and psychopharmacology may be making only a small contribution to improving the quality of life of patients with schizophrenia. Although the impact of schizophrenia affects a smaller proportion of the global population than depressive and anxiety disorders, the weight of disability during the acute psychotic state of schizophrenia was speculated to be the highest across in the Global Burden of Disease Study 2019 [1,2]. Notably, unlike depressive or anxiety disorders, variations in schizophrenia are not observed across regions or sexes, with disability continuing into old age [1]. 

Following the successful discovery of typical antipsychotics, such as phenothiazines and butyrophenones, the scientific basis for these medications was summarized in the dopamine hypothesis of schizophrenia [3,4,5,6,7,8]. The dopamine hypothesis has been repeatedly/continuously revised, and the majority of psychiatrists have been convinced that appropriate pharmacological therapies have contributed to re-integrating patients with schizophrenia into society [9]. However, in the 1980s, psychiatrists recognized that the major disability in schizophrenia consists of not only positive symptoms, which are the primary targets of typical antipsychotics but also cognitive impairments and negative symptoms, which are less responsive to typical antipsychotics [10,11,12]. Typical antipsychotics relieve positive symptoms, such as hallucinations and delusions, via the inhibition of the dopamine D2 receptor. However, typical antipsychotics are not only ineffective for treating negative symptoms and cognitive impairments but also the potential causes of their exacerbation [13,14]. Psychiatry and psychopharmacology improved the dopamine hypothesis to develop more effective antipsychotics and dialed other monoamine receptor targets into the basic dopamine D2 mechanism, including the antagonism of serotonin receptors, to develop atypical antipsychotics (the revised dopamine hypothesis) [15,16].

Despite the increasing number of approved antipsychotics, several meta-analyses that compared typical and atypical antipsychotics revealed no significant differences in their efficacy, with the exception of clozapine, which is significantly more effective than other conventional antipsychotics [13,17]. Based on this historical background and the insufficient efficacy of conventional antipsychotics for the treatment of negative symptoms and cognitive impairments, alternative approaches to developing novel antipsychotics involving targets other than monoamine receptors have been attempted. Unfortunately, psychiatry has yet to produce novel antipsychotics with major targets other than monoaminergic transmission. However, the successful development of several novel types of antipsychotics is becoming more realistic, including KarXT (muscarinic acetylcholine receptor agonist) [18,19,20,21], ulotaront (agonist of trace-amine-associated receptors) [22,23], and iclepertin (glycine-transporter inhibitor) [24,25].

Large-scale genome-wide association studies (GWAS) identified over 100 risk genes for schizophrenia. Notably, approximately 30% of them encoded proteins that are associated with glutamatergic transmission [26,27,28]. For some time, it has been well-known that patients with schizophrenia can be resistant to treatment with typical and atypical antipsychotic medications [29,30]. Clozapine, the sole approved antipsychotic for patients with treatment-resistant schizophrenia (TRS) [31,32,33], has a lower affinity for the dopamine D2 receptor in comparison to conventional typical/atypical antipsychotics and enhances glutamatergic transmission [34,35,36,37,38]. These accumulating findings from psychopharmacological, neurobiological, and molecular biology studies suggest that the core of the pathophysiology and/or pathomechanism in a proportion of patients with schizophrenia probably involves the dysfunction of N-methyl-D-aspartate glutamate receptors (NMDARs). Despite intensive research, glutamatergic antipsychotics, according to the glutamate hypothesis, have not been approved for the treatment of schizophrenia to date. However, considering the increasing clinical and preclinical findings supporting the potential pro-cognitive effects of glutamatergic agents and the lack of sufficient treatment for the negative symptoms or cognitive impairments associated with schizophrenia, these previous setbacks cannot preclude research into potential glutamate modulators as novel antipsychotics. Given this background, to emphasize the importance of the glutamate hypothesis, this review introduces the increasing findings regarding functional abnormalities in glutamatergic transmission associated with the NMDAR in schizophrenia and/or TRS. Additionally, this review also mentions a new challenge in psychiatry, namely ultra-treatment-resistant schizophrenia (UTRS) [29,30,39]. UTRS is a recently proposed concept characterizing the specific clinical features of clozapine resistance [29,30,39]. However, the actual pathomechanism of UTRS is still unknown, as the actual pathophysiology of clozapine currently remains to be clarified.

## 2. NMDAR Function and Modulation

**Figure 1:**
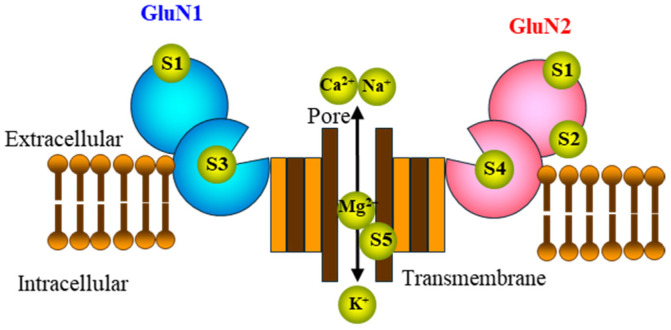
Schematic structure of the N-methyl-D-aspartate glutamate receptor (NMDAR). NMDAR is a heterotetrameric complex with GluN1, GluN2, and GluN3 subunits. S1: glutathione binding site (in GluN1 and GluN2A). S2: Zn^2+^ binding site (Zn^2+^: in GluN2A and ifenprodil/Ro25-6981 in GluN2B). S3: glycine binding site in GluN1 (glycine, D-serine, and kynurenic acid). S4: L-glutamate binding site in GluN2. S5: phencyclidine binding site in transmembrane domain (MK801, ketamine, and phencyclidine).

Glutamate is the most predominant excitatory transmitter in the brain. Glutamate receptors are categorized as ionotropic or G-protein-coupled metabotropic receptors. Ionotropic glutamate receptors are further divided into three subtypes based on their ligands, namely N-methyl-D-aspartate (NMDAR), α-amino-3-hydroxy-5-methylisoxaole-4-propionate (AMPAR), and kainic acid (KAR) receptors. Ionotropic glutamate receptors are tetramers (composed of four subunits). The majority of AMPARs and KARs are mainly permeable to sodium and potassium, but calcium permeability is energetically unfavorable. However, among ionotropic glutamate receptors, NMDARs can allow the inflow of sodium, potassium, and calcium, depending on the plasma-membrane potential, and exhibit slower and incomplete desensitization in comparison to AMPARs and KARs [40]. Furthermore, the penetration of calcium through NMDAR affects several signalings as a second messenger [41]. Because of these cation permeabilities, ionotropic glutamate receptor hyperactivation contributes to neurotoxicity. Therefore, the appropriate modulation/activation of ionotropic glutamate receptors, including the NMDAR, requires pharmacological manipulation that does not induce NMDAR neurotoxicity. A schematic of the NMDAR structure is displayed in Figure 1 to explain its various pharmacological modulatory sites.

The NMDAR is composed of a heterotetrameric complex with two GluN1 and two GluN2 subunits or two GluN1, one GluN2, and one GluN3 subunit. Several ligand binding sites for regulating ion channel activity and their selective ligands have been identified, such as the glutamate binding site in GluN2 (L-glutamate), the glycine modulatory binding site in GluN1/GluN3 (D-serine, glycine, and kynurenic acid), the Zn^2+^ binding site in GluN2A/GluN2B (Zn^2+^, ifenprodil, and Ro25-6981), the redox-regulating glutathione binding site (in GluN1/GluN2A), the spermine binding site in GluN2B, and the phencyclidine binding site in the transmembrane domain (Mg^2+^, MK801, ketamine, and phencyclidine) [41,42,43,44,45]. The activation of the NMDAR requires three events, namely the removal of Mg^2+^ from the transmembrane domain (Mg^2+^ blocks the cation channel pore) due to postsynaptic depolarization (usually due to activated AMPAR), the binding of glycine/D-serine to the glycine modulatory site in GluN1, and the binding of L-glutamate to the glutamine binding site in GluN2 [40]. In other words, the NMDAR has unique gating modes, namely ligand gated and voltage gated.

To date, numerous enhancers of ionotropic glutamate receptors have been synthesized [46,47]. The expression patterns of NMDARs seem to be regulated by the GluN2 subunit [48,49,50,51]. GluN2A is highly expressed in the hippocampus/cortex and modestly expressed in the midbrain, cerebellum, striatum, and brainstem [48], and GluN2B is predominantly expressed in the hippocampus, cortex, and striatum [49]. GluN2C is mainly expressed in the cerebellum and thalamus [50], and GluN2D is primarily expressed in the brainstem and forebrain [51]. Based on these expression patterns, positive allosteric modulators (PAMs) of GluN1/GluN2A were developed [46].

A positive allosteric modulator (PAM) is a ligand that enhances the function of a receptor in the presence of an agonist but is unable to directly activate the receptor without the agonist. PAMs can increase NMDAR activity by enhancing the binding of endogenous agonists to NMDARs. Therefore, PAMs are considered to have broader therapeutic applications than agonists due to the lower risk of neurotoxicity induced by NMDAR hyperactivation. First-generation NMDAR PAMs have been pursued as therapeutics, as NMDAR hypofunction has been identified in multiple brain diseases. Several NMDAR PAMs have entered clinical trials, providing human data on the effects of NMDAR potentiation. CAD-9303 has been studied in patients with schizophrenia (NCT04306146). In addition, AGE-718 is being studied in people with Huntington’s disease (NCT05358821 and NCT05107128), Parkinson’s disease (NCT05318937), and Alzheimer’s disease (NCT04602624).

Based on the expression patterns of NMDARs and the advantages of PAMs, positive allosteric modulators of GluN1/GluN2A have been developed, such as thienopyrimidin-4-ones, thiazole pyrimidinone, benzofuran analogs, and benzohydrazide analogs [46]. In order to proceed to clinical trials, expanding the structural diversity of lead compounds, enhancing pharmacodynamic features by optimizing the balance between water solubility and lipophilicity to improve bioavailability, and improving selectivity toward individual GluN2 subunits should be achieved [46,47,52].

## 3. Clinical Findings Supporting the Glutamate Hypothesis

### 3.1. Initial Clinical Findings

The most impactful initial clinical findings were observed in individuals who abused the NMDA antagonists phencyclidine and ketamine [53,54]. Both phencyclidine and ketamine produce not only positive symptoms (including auditory hallucinations) but also negative symptoms and cognitive impairments for up to 2 weeks [54]. Furthermore, the intake of phencyclidine and ketamine in patients with schizophrenia exacerbates its symptoms [55,56]. It was reported that 6.5% of patients who fulfilled the diagnostic criteria for schizophrenia at the first episode of schizophrenia-like psychosis were positive for anti-NMDAR antibodies [57]. A meta-analysis study of seven reports also demonstrated that anti-NMDAR antibodies were detected in 8% of patients with schizophrenia (115/1441) [58]. Furthermore, a recent OPTIMISE project also reported that approximately 5% of first-episode psychosis patients without antipsychotic exposure were positive for anti-NMDAR antibodies [59]. Notably, the serum levels of anti-NMDAR antibodies in first-episode patients with schizophrenia were positively correlated with the severity of schizophrenia using PANS scores [60]. These clinical findings suggest that the psychotic symptoms induced by NMDAR antagonists and anti-NMDAR autoantibodies are similar to those of schizophrenia [61].

NMDAR antagonists directly inhibit the permeability of channel pores, whereas NMDAR antibodies have no direct effect on channel-pore function [62]. However, anti-NMDAR antibodies lead to the internalization/downregulation of NMDARs [59,63,64,65]. These preclinical findings suggest that decreasing NMDAR activity by either decreasing functional NMDARs in the plasma membrane or directly inhibiting channels plays an important role in the negative symptoms and cognitive impairments caused by schizophrenia. Based on clinical and preclinical findings, encephalitis induced by anti-NMDAR antibodies has been established as an autoimmune disorder with schizophrenia-like symptoms [63]. Clinical findings showing that NMDAR antagonists and anti-NMDAR antibodies lead to schizophrenia-like symptoms contributed to the development of the glutamate hypothesis of the pathophysiology and pathomechanisms of schizophrenia.

The NMDAR antagonist esketamine is approved for treatment-resistant depression, though not for schizophrenia, and is a more rapid-acting antidepressant in comparison to monoamine-transporter inhibitors [66]. Anecdotal information about ayahuasca, which is a psychedelic plant brew originating from the Amazon rainforest, varies significantly, ranging from evangelical accounts to horror stories involving physical and psychological harm [67]. The antidepressive effects of ayahuasca were first reported in 1984, and a candidate mechanism underlying its antidepressive action was speculated to be monoamine oxidase inhibition [68]. A systematic review also highlighted the antidepressive effects of ayahuasca. However, the review was limited by small sample sizes and a lack of healthy controls [69]. Harmine, which is one of the abundant β-carboline alkaloids in ayahuasca, enhances glutamate uptake by increasing glutamate-transporter expression in animal models [70,71]. In addition, clinical evidence shows that the partial NMDAR agonist D-cycloserine induces a depressive mood [72]. Therefore, attenuating glutamatergic transmission, likely ketamine/esketamine and ayahuasca, plays an important role in the improvement of a depressive mood.

### 3.2. Post Mortem Brain Studies

Post mortem brain studies have shown variable changes in the expression of NMDARs (protein and mRNA) and some of the postsynaptic molecules associated with NMDAR signaling in patients with schizophrenia. Both the mRNA and protein expressions of the GluN1 and GluN2C subunits in the prefrontal cortices of patients with schizophrenia were significantly decreased in comparison to that in healthy controls, whereas changes in the other subunits, such as GluN2A, GluN2B, GluN2D, and GluN3, in the prefrontal cortex were not observed [73,74]. Decreased expression of GluN1 mRNA in the dentate gyrus of patients with schizophrenia compared to controls was also reported [75]. Furthermore, the expression of several NMDAR-related postsynaptic density molecules was also altered in the post mortem brains of patients with schizophrenia. The protein and mRNA expressions of both postsynaptic density protein-93 (PDS-93) and PDS-95 were decreased in the anterior cingulate cortex [73,76,77,78]. Other studies also revealed altered levels of glutamate, N-acetylaspartylglutamate, kynurenic acid, and the activity of glutamate carboxypeptidase II in schizophrenia [79,80,81,82] (N-acetylaspartyl-glutamate, which is degraded by glutamate carboxypeptidase II, is an endogenous NMDAR antagonist and mGlu3R agonist [83,84]). Increased levels of endogenous NMDAR antagonists, N-acetylaspartyl-glutamate and kynurenic acid [85,86,87], and/or the decreased activity of glutamate carboxypeptidase II in schizophrenia may support the hypothesis that N-acetylaspartyl-glutamate signaling is involved in NMDAR hypofunction in schizophrenia [79,80,81,82,83,84].

### 3.3. Genome-Wide Association Studies

Over the past 15 years, several large-scale genome-wide association studies (GWASs) of schizophrenia have been conducted; the largest-scale GWAS included over 76,000 patients with schizophrenia and 240,000 healthy controls [26,27,28]. Most of the identified single-nucleotide polymorphisms (SNPs) associated with schizophrenia were located in non-coding regions. However, notably, approximately 30% of them encoded proteins related to glutamatergic transmission [27,88]. Copy-number variants (CNVs), in which kilobases of DNA are deleted or repeated, linked to the risk of schizophrenia have been found to be enriched with gene-encoding proteins associated with glutamatergic synapses, and the postsynaptic density has also been implicated [89]. Fine-mapping/exome sequencing also identified coding variants associated with substantial risks for schizophrenia in the NMDAR (GluN2A), the AMPAR (GluA3), the KAR (GluK2 and GluK4), and PSD-95-associated genes (SHANK, NLGN, and DLGAP) and serine racemase (SR) [90,91].

### 3.4. Neurophysiological Findings

Proton magnetic resonance spectroscopy (MRS) and positron emission tomography (PET) enable the non-invasive visualization of the brain and the measurement of functions of numerous transmitters, including dopamine and glutamate, in living human subjects in vivo. Regional cerebral blood flow in the frontal cortices of patients with schizophrenia was predominantly increased by ketamine administration compared to healthy controls, as observed using PET [92]. An MRS study showed increased glutamatergic transmission in the limbic system and basal ganglia, glutamine levels in the thalamus, and levels of glutamate plus glutamine in the basal ganglia and medial temporal cortex [92,93,94,95,96]. Additionally, ketamine administration in healthy controls caused an acute increase in glutamine that was marginally related to cognitive function [97,98]. These findings possibly support the notion that people with schizophrenia are hypersensitive to NMDAR antagonists compared to healthy controls.

### 3.5. Psychophysiological Findings

Event-related potentials detected using both electroencephalography and magnetoencephalography enable the high-resolution temporal monitoring of physiological brain function. Mismatch negativity and P300 are considered to be indices for auditory sensory memory/pre-attentive processing and re-orienting/covert attention shifting, respectively [98,99,100]. Currently, reductions in the mismatch negativity and P300 amplitude are both hypothesized to reflect NMDAR hypofunction and are considered candidate biomarkers for schizophrenia [101,102]. Indeed, the administration of ketamine is known to attenuate the mismatch negativity and P300 amplitude in healthy subjects [103,104]. A reduction in the mismatch negativity amplitude has also been observed in patients with schizophrenia [105,106].

## 4. Preclinical Findings Supporting the Glutamate Hypothesis

### 4.1. Genetic Animal Model

Exploratory studies of functional abnormalities in genetic animal models with the knockout of serine racemase (SR-KO) have contributed to the acquisition of numerous findings about the pathophysiology and pathomechanisms of schizophrenia. SR, which converts L-serine to D-serine [107], is one of the risk genes for schizophrenia identified by GWASs [26,27,28]. Therefore, the findings of functional abnormalities in SR-KO can be considered as a “proof of principle” that glutamatergic transmission is a candidate therapeutic target for schizophrenia.

SR−KO exhibits a reduction in cortical D-serine (90%) and NMDAR-sensitive long-term potentiation (70%) [108,109,110]. Various behavioral abnormalities associated with schizophrenia have also been detected, such as impairments in memory components and hyperactivity [109,110,111,112]. A quantitative MRI study detected cortical atrophy (5%) and increased ventricle volume (20%) [113]. Golgi staining demonstrated decreased dendritic complexity and synaptic spine density in several cortical regions and the hippocampus [109,111,114], suggesting the loss of approximately 30% of cortical glutamatergic synapses [114,115]. Additionally, decreased parvalbumin in cortical GABAergic neurons and perineural nets surrounding the GABAergic neurons were also observed in SR-KO [116]. The normalization of D-serine levels in the brain induced by the chronic administration of D-serine improved some of the observed abnormalities in SR-KO, such as long-term potentiation, the reversal of memory deficits, the correction of cortical neurochemical abnormalities, and the partial restoration of dendritic spines [109,117]. Therefore, these findings suggest that SR-KO is a pharmacologically valid candidate genetic animal model [118] for schizophrenia [88]. Considering that the NMDAR requires three events for activation, namely the depolarization of the plasma membrane, the binding of D-serine/glycine to the glycine modulatory site in GluN1, and the binding of L-glutamate to the glutamine binding site in GluN2 [40], the glycine modulatory site in GluN1 plays an important role in the pathophysiology of schizophrenia or cognition.

Recently, several functional abnormalities, such as hypoactivity in the mPFC, increased dopaminergic signaling in the striatum, hypersensitivity to amphetamine-induced hyperlocomotion, fewer molecules associated with glutamate receptors in the glutamatergic synapse, and aberrant locomotor patterns opposite to those associated with antipsychotic-induced behavior, were observed in GluN2A knockout mice (GluN2A-KO) [119]. These findings from two genetic animal models, SR-KO and GluN2A-KO, suggest that glutamatergic transmission hypofunction plays an important role in the pathomechanisms of schizophrenia.

### 4.2. Pharmacological Animal Model

The systemic administration of NMDAR antagonists (phencyclidine, ketamine, and MK801) increased the release of dopamine [120,121], serotonin [121,122], and norepinephrine [123,124] in the mPFC. Local NMDAR antagonist administration into the mPFC also increased the release of dopamine, serotonin, and norepinephrine in the mPFC [121,125,126,127], but these increases in monoamine release in the mPFC were inhibited by the local administration of muscimol (a GABAA receptor agonist) into the mPFC [121,125,126,127]. These findings suggest that the presynaptic terminals of monoaminergic projections are subject to GABAergic inhibition, which is positively regulated by the NMDAR. Furthermore, the local administration of an NMDAR antagonist into the ventral tegmental area (VTA), locus coeruleus (LC), and dorsal raphe nucleus (DRN) also increased the release of dopamine, norepinephrine, and serotonin in the mPFC. These increases in monoamine release in the mPFC were also inhibited by the local administration of muscimol into the VTA, LC, and DRN [120,125,126,127,128,129,130,131]. Therefore, monoaminergic neurons in the VTA, LC, and DRN are also subject to GABAergic inhibition, which is positively regulated by the NMDAR, similar to the mPFC.

Similar to monoaminergic systems, systemic NMDAR antagonist administration also increased the release of L-glutamate in the mPFC [120,128,132]. However, the local administration of neither the NMDAR antagonist nor muscimol into the mPFC affected L-glutamate release in the mPFC [120,121,129,132]. In contrast, the local administration of an NMDAR antagonist into the mediodorsal (MDTN) and reticular thalamic nuclei (RTN) drastically increased L-glutamate release in the frontal cortex [120,122,131]. A detailed analysis of the transmission pathway among the RTN, MDTN, and mPFC indicated that the RTN projects GABAergic terminals into the MDTN (but not the mPFC), and the MDTN projects glutamatergic terminals into the mPFC [66,120,122,131,133,134]. These observations suggest that an impaired NMDAR function in the RTN leads to a GABAergic disinhibition of glutamatergic neurons in the MDTN, resulting in increased glutamate release in the mPFC. 

These results indicate that the attenuation of the NMDAR leads to the increased transmission of monoamine and glutamate in the mPFC, but the mechanisms of monoaminergic and glutamatergic transmission in the mPFC are not identical. Notably, thalamocortical glutamatergic transmission is mainly regulated by intrathalamic GABAergic transmission, which is enhanced by the activation of thalamocortical glutamatergic transmission via NMDARs in the RTN [124,131,135,136]. Interestingly, monoaminergic inputs to the RTN are positively regulated via excitatory 5-HT7 receptors and α1 adrenoceptors [124,131,135,136]. 

## 5. Treatment-Resistant Schizophrenia and Limitation of Conventional Antipsychotics

### 5.1. Treatment-Resistant Schizophrenia (TRS)

Treatment-resistant schizophrenia (TRS) and the limitations of conventional antipsychotics are major issues in psychiatry. Since the approval of atypical antipsychotics, this topic has become more actively discussed [137,138,139]. The concept of TRS is tied to two clinical issues, namely a lack of improvement in psychotic episodes in patients with schizophrenia and criteria for initiating clozapine medication. Several studies have provided important clinical insights into TRS. Patients with TRS are more often of European descent [140,141] and affected by the paranoid subtype [141,142]. Compared with non-TRS, the onset age was younger [141,142,143,144], and premorbid social functioning was poorer in TRS [145]. The largest population-based cohort study to identify the clinical features of TRS was based on Danish national registry data, which were used to compare patients with TRS to all other patients diagnosed with schizophrenia over a ten-year period [142]. This study found that individuals with TRS were more likely to have a comorbid personality disorder, a more rural residence, more schooling, and a previous history of suicide attempts [142]. At the time of first schizophrenia diagnosis, patients with TRS were more likely to be inpatients, to have required more psychotropic medications in the previous year, and to have spent more than 30 days in the psychiatric hospital in the previous year [142].

Defining TRS, which means conventional antipsychotic-resistant schizophrenia, has been a challenge for the field, and numerous guidelines with potential criteria for TRS have been published [29,30,146,147] (Table 1). The Treatment Response and Resistance in Psychosis (TRIPP) Working Group provided the newest consensus criteria for TRS and guidelines for the treatment of TRS (Table 1) [29]. The TRIPP group reported that 95% of the previous studies adopted different criteria to define TRS (Table 1), 50% reported operationalized criteria, and 60% required that patients had not responded to at least two adequate treatment trials and defined adequate treatment as lasting for at least 6 weeks, the typical time for a medication response. However, 50% of these studies did not report the dosage used but instead stated only “adequate dose” [29]. These results emphasize the need for more rigorous and standardized criteria for TRS.

### 5.2. Ultra-Treatment-Resistant Schizophrenia (UTRS)

Traditionally, more than 30% of patients with schizophrenia are considered to have TRS [29,30]. Clozapine is the sole approved antipsychotic for TRS, owing to its higher efficacy in the treatment of TRS in comparison with typical antipsychotics (approximately 30–60% of patients with TRS can respond to clozapine [31,32,33]). Based on these figures, the criteria for defining TRS are currently recognized as useful for the indication of clozapine and are utilized as such.

The first study to demonstrate the superiority of clozapine over other antipsychotics was reported by Kane, and this report contributed to the approval of clozapine for the treatment of TRS [31]. This study revealed that 30% of haloperidol-resistant patients were improved by clozapine, but chlorpromazine improved 4% of patients [31]. Subsequently, several meta-analyses also confirmed the superiority of clozapine over other typical antipsychotics [32,33]. Two large-scale prospective effectiveness studies (CATIE and CUtLASS) also revealed the superiority of clozapine for TRS over atypical antipsychotics [148,149]. A recent network meta-analysis contradicted the evidence of the superiority of clozapine compared to other antipsychotics [150]. The discrepancy in the evaluated effectiveness of clozapine between the large-scale prospective effectiveness studies (CATIE and CUtLASS) [148,149] and the meta-analysis [150] was explained by two possibilities, namely sampling bias and the fact that the meta-analysis study did not include the data in CATIE or CUtLASS [151].

However, it is notable that evidence shows that over 30% of patients with TRS also exhibit clozapine resistance [31,152,153]. Recently, the TRIPP group proposed the term ultra-treatment-resistant schizophrenia (UTRS) due to the specific features of clozapine resistance [29,30,39]. To understand the basis of UTRS, a more detailed scientific analysis is needed.

### 5.3. Primary TRS and Secondary TRS

Clinically, the failure to respond to appropriate treatment with more than two antipsychotics is regarded as a fundamental criterion for TRS [29,30,146,147]. Recently, it has been argued that two factors may be involved in the pathomechanism or pathophysiology of TRS [154]. Specifically, TRS is defined as primary or secondary. In primary TRS, the antipsychotic-resistant pathology already exists at the first episode of schizophrenia, and the pathophysiology of secondary TRS is augmented by persistent adequate antipsychotic medications [155,156,157].

The hyperactivation of dopaminergic transmission, including the supersensitivity and upregulation of dopamine D2 receptors, induced by consecutive exposure to D2 receptor antagonistic antipsychotics is the basis for secondary TRS [154,158]. The overall response to all antipsychotics was reported to be 40–60% [159,160]. The response to antipsychotics in antipsychotic-naïve individuals is estimated to be approximately 75%. However, the response to a second trial of antipsychotics other than clozapine is considerably lower, ranging from 20 to 45% [161,162]. These discrepancies in response rates between the first and second trials of antipsychotic medications suggest the possibility that consecutive exposure to D2 receptor antagonists plays a fundamental role in the development of antipsychotic resistance. Indeed, the maximum response rate to a clozapine trial was 80% when treatment was initiated within the first 2–3 years after resistance was established, whereas, when resistance had been established for a longer period of time, the response rate decreased to approximately 30% [160,161,163].

The affinity of clozapine for the D2 receptor is markedly lower than that of conventional typical/atypical antipsychotics [154,164]. Additionally, the rapid dissociation of clozapine from D2 receptors has been considered a candidate mechanism for the decreased supersensitivity of the D2 receptor induced by clozapine [134,164,165,166]. However, the dissociation rate of clozapine from D2 receptors is not significantly faster than those of other antipsychotics, such as quetiapine, amisulpride, remoxipride, and sulpiride [164,167,168,169]. Rather, several antipsychotics act as pharmacological chaperones to D2 receptors independent of dopaminergic signaling, which contributes to the upregulation of D2 receptors. However, the impact of clozapine on D2 receptor trafficking to the plasma membrane is the lowest among antipsychotics [170]. Furthermore, in a recent study, clozapine did not upregulate numerous monoamine receptors via the suppression of protein phosphatase 2A, which regulates the turnover of monoamine receptors [38]. These clinical advantages of clozapine for secondary TRS over other atypical antipsychotics are likely related to the distinct pharmacological profile of this drug.

## 6. Novel Treatment Based on Glutamate Hypothesis

Both clinical and preclinical findings indicate the possibility that NMDAR modulation can provide novel treatments for TRS. In particular, the finding that 30% of risk genes for schizophrenia identified by GWASs affect NMDAR-associated signaling has attracted attention, as this suggests that, in a proportion of patients with schizophrenia (approximately 30%), the pathogenesis can be explained by the glutamate hypothesis [26,27,28]. Given that the prevalence of primary TRS is also approximately 30% [31,152,153], antipsychotics targeting the NMDAR are attractive novel candidates for the treatment of schizophrenia. To avoid excitotoxicity induced by the activation of NMDARs, the modulation of the glycine modulatory site in GluN1 (but not the direct activation of the glutamate binding site in GluN2) was adopted as a candidate approach [84,171].

### 6.1. D-Serine and D-Cycloserine

Both D-serine and glycine are endogenous agonists at the glycine modulatory site in GluN1 [40,172]. The activation of the NMDAR requires the binding of L-glutamate to the glutamate binding site in GluN2, accompanied by the binding of D-serine or glycine to the glycine modulatory site in GluN2. D-cycloserine is a non-endogenous partial agonist at the glycine modulatory site with lower efficacy (approximately 50% intrinsic affinity) than endogenous agonists [40,172].

Most double-blind placebo-controlled studies of D-serine and D-cycloserine have demonstrated their effects on negative symptoms and their modest effects on cognition [173], since adjunctive D-cycloserine was effective in addressing all three symptom clusters [174,175]. However, the results of subsequent trials on D-cycloserine efficacy were not consistent. Indeed, some trials found it to be ineffective [176,177]. The therapeutic window of D-cycloserine is speculated to be extremely narrow [178]. A moderate dose/concentration of D-cycloserine, which enhances cognition in experimental animals [179], can activate the NMDAR, but at high doses, D-cycloserine acts as an antagonist due to its low intrinsic efficacy [40,172].

Despite their efficacy, these agents have several shortcomings that render them unsuitable for long-term administration in patients with schizophrenia, such as adverse reactions (renal tubular necrosis, type 2 diabetes mellitus, and heart-valve disease) and a reduction in their efficacy induced by the desensitization of the NMDAR, resulting in the loss of their efficacy [180,181,182,183,184,185].

### 6.2. Glycine-Transporter Inhibitors

The glycine transporter, composed of SC6A5 and SLC6A9, is known to be a reversible transporter with Na^+^/Cl^−^ dependence. However, with substantial intracellular glycine accumulation, the glycine transporter conversely releases glycine into the extracellular space [24,186]. Therefore, the inhibition of the glycine transporter increases glycine levels in the synaptic cleft, resulting in the activation of the NMDAR. An endogenous glycine transporter inhibitor, sarcosine, is produced in the glycine synthesis pathway [187,188]. Sarcosine application can enhance NMDAR activity as a glycine source via either reverse uptake or heteroexchange [189]. Several randomized control studies of adjunctive sarcosine to antipsychotics demonstrated its efficacy in the treatment of positive and negative symptoms compared to placebo and D-serine intake groups [176,188,190,191]. However, sarcosine and its derivatives lead to several neurological adverse reactions, such as hypoactivity and ataxia [192].

Randomized control phase 2 studies of iclepertin (adjunct to antipsychotics) demonstrated that iclepertin improved cognitive functioning compared to placebo-treated controls [193,194]. However, the results of a series of phase 3 trials in the “CONNEX” program and long-term open-label studies remained to be published [195]. None of the studied agents have successfully completed clinical trials. However, the recent encouraging results of phase 2 studies of iclepertin have reinvigorated the exploration of NMDAR modulators as effective treatment options for schizophrenia.

## 7. TRS and Clozapine

### 7.1. Impact of Clozapine on Glutamatergic Agents

Most trials reported that adjunctive D-serine, D-cycloserine, and glycine-transporter inhibitor with clozapine were negative [196,197,198]. Notably, adjunctive D-cycloserine with clozapine and risperidone worsened and improved negative symptoms, respectively [198,199]. Preclinical studies reported that clozapine activated NMDAR via the enhancement of glutamatergic transmission. Clozapine increased the extracellular levels of L-glutamate, D-serine, and glycine through the enhancement of neuronal exocytosis, astroglial non-exocytosis, and the inhibition of the glycine transporter [34,35,36,37,38]. The other actions of clozapine have been reported, including that clozapine enhances occupancy in the glycine-modulating site [200], and interacts with metabotropic glutamate receptors [38,154,201,202,203,204]. The administration of D-cycloserine, D-serine, and glycine-transporter inhibitors in experimental animals improved the memory disruptions induced by MK801 and phencyclidine [187,205,206,207,208,209]. Notably, the co-administration of a glycine-transporter inhibitor and antipsychotics other than clozapine enhanced glutamatergic transmission but attenuated dopaminergic side effects [207].

Considering these preclinical findings, clozapine enhances cognition via the activation of both glutamate and glycine binding sites by increasing the extracellular levels of L-glutamate and D-serine [133,202]. In the absence of clozapine, D-cycloserine can enhance NMDAR function via the activation of the glycine binding site, but under the increasing extracellular D-serine level induced by clozapine, D-cycloserine conversely may suppress the glycine binding site due to its low intrinsic partial agonistic action (approximately 50%), resulting in inhibiting the stimulatory effects of clozapine on NMDAR function. In conclusion, D-cycloserine can improve negative symptoms (approximately 10–26%) and a part of cognitive dysfunction (working memory and experience-dependent neuroplasticity) in patients with schizophrenia [210]. However, adjunctive D-cycloserine to clozapine not only probably inhibits the clinical action of clozapine but also worsens them.

The accumulating pharmacodynamic findings on clozapine support that clozapine may be an antipsychotic agent and has a mechanism of action that corresponds to both revised dopamine and glutamate hypotheses. However, the serious/lethal adverse reactions to clozapine, such as metabolic, hematologic, and neurological complications; cardiotoxicities; and discontinuation syndromes, are more severe than recognized by general psychiatrists [133,202,211,212,213,214,215,216]. Therefore, in the future, it will be important to establish antipsychotic medication that overcomes the double-edged sword features of clozapine.

### 7.2. Novel Pharmacological Targets of Clozapine

Clinical findings for both clozapine and NMDAR enhancers (glycine-transporter inhibitor, D-serine, and D-cycloserine) suggest that the vulnerability of glutamatergic transmission probably plays an important role in the pathomechanism of a proportion of patients with TRS. The clinical finding is that the adjunctive administration of NMDAR enhancers in patients treated with clozapine had no effect or worsened the condition indicates that clozapine enhances glutamatergic transmission, but at the same time, it also suggests that it may have action targets other than enhanced glutamatergic transmission. Recently, we reported that a candidate gliotransmitter, L-BAIBA, may contribute to the mechanism underlying several adverse reactions to clozapine that could not be explained by the receptor binding profile of clozapine alone [38,154,202,203].

L-BAIBA is an isomer of GABA and is structurally similar to GABA [202]. Indeed, L-BAIBA binds to both GABA-A and GABA-B receptors [154,217]. Furthermore, L-BAIBA also binds to Mas-related G-protein-coupled receptor type D (MRGPRD), the glycine receptor, and III-GluR [154,218]. L-BAIBA increases AMP-dependent kinase (AMPK) signaling by activating MRGPRD, leading to metabolic complications [203]. The activation of AMPK signaling induced by L-BAIBA suppresses protein phosphatase 2A (PP2A) activity, resulting in the inhibition of the upregulation of dopamine D2, serotonin 5-HT1A, 5-HT2A, and 5-HT7 receptors (downregulation in animal models) [38]. The suppressive effects of clozapine on PP2A through an increase in L-BAIBA have been proposed to play an important role in serotonergic clozapine-discontinuation syndrome/withdrawal syndrome [38]. The discovery that clozapine inhibits PP2A activity by increasing L-BAIBA signaling contributed to the revision of the traditional pathophysiological hypothesis of serotonergic clozapine-discontinuation syndrome [38]. Similarly, a pathophysiological hypothesis of clozapine-discontinuation catatonia can be constructed via the effects of L-BAIBA on PP2A and GABA-B receptors [38,154,202]. These pathophysiological hypotheses of L-BAIBA can reasonably explain the time lag in the onset of serotonergic symptoms and catatonia after clozapine discontinuation (they usually appear 1–2 weeks after clozapine discontinuation) [38]. Thus, the mechanisms underlying the clinical efficacy and adverse reactions of clozapine, which cannot be fully explained by its receptor binding profile, may be mediated by novel molecules whose functions are induced by clozapine [38,133,154,202]. Considering the clinical efficacy and severe/lethal adverse reactions of clozapine, exploring and discovering novel targets of clozapine may contribute to the development of novel treatments for schizophrenia with fewer adverse reactions and a clinical efficacy comparable to that of clozapine. Although the pathomechanisms of the recently proposed UTRS (clozapine-resistant schizophrenia) [29,30,39] are speculated to involve functional abnormalities other than glutamatergic transmission, it should be noted that, unless we identify the actual mechanisms and unknown targets regarding the action of clozapine, the interpretation of hypothetical pathophysiology and/or pathomechanisms of UTRS may be off the mark.

## 8. Conclusions

This review discusses the attractive viewpoints of the glutamate hypothesis as a background for the development of novel antipsychotics independent of the dopamine hypothesis; part of the discussion includes the possibility that patients with glutamatergic dysfunction may be one of the major subgroups of TRS. However, considering the results of GWASs, patients with schizophrenia with glutamatergic dysfunction or acquired risks related to glutamatergic transmission are speculated to account for approximately 30% of all individuals with schizophrenia. Therefore, the population of patients with schizophrenia is probably composed of individuals resistant to not only dopaminergic agents but also glutamatergic agents or both (resistant to dopaminergic and glutamatergic agents). In other words, when evaluating the efficacy of novel glutamatergic agents through randomized controlled trials in the entire population of patients with schizophrenia, the results may be skewed due to individuals who are sensitive to dopaminergic antipsychotics but insensitive to glutamatergic agents. Furthermore, because of severe adverse reactions to clozapine, which is currently the sole approved antipsychotic for the treatment of TRS, it is important to strive to develop novel antipsychotics that are more effective in the treatment of TRS than conventional antipsychotics, are safer than clozapine, and can prevent the development of secondary TRS. Considering the pharmacological features of clozapine (increasing extracellular levels of endogenous NMDAR agonists), second-generation NMDAR PAMs may be candidate agents that are clinically efficacious in addition to clozapine. Finally, the development of treatment for UTRS will be the most important challenge in psychiatry in the future. Thus, pharmacodynamic analyses to clarify the unknown pathophysiology of clozapine are becoming increasingly important.

## Figures and Tables

**Table 1:** Proposed Defining Treatment-Resistant Schizophrenia.

	Treatment Failure	Duration	Failure Criteria
APA (2004)	>2 >1 (atypical)	>6 weeks	Little/no response to adequate duration/dose (therapeutic range)
NICE (2014)	>2 (Sequential) >1 (no clozapine atypical)	4–6 weeks	Little/no response to adequate dose and correct duration (despite established adherence to medication)
WFSBP (2012)	>2 unidentical classes >1 (atypical)	2–8 weeks	No significant improvement in the psychopathology and/or target symptoms (ensured treatment adherence)
TRRIP (2017)	>2 (atypical) >1 (LAI > 4 Month)	>6 weeks (therapeutic dose)	More than moderate severity <20% symptom reduction during prospective or observation of >6 weeks At least moderate functional impairment based on a validated scale adherence (>80% of prescribed doses) confirmed by serum levels

Abbreviations: APA: American Psychiatric Association [30], NICE: National Institute for Health and Care Excellence [146], TRRIP: Treatment Response and Resistance in Psychosis [29], WFSBP: World Federation of Societies of Biological Psychiatry [147]. LAI: long-acting injectable antipsychotics.
